# Comparing the effectiveness of animated videos and talking‐head videos in science communication

**DOI:** 10.1111/bjhp.12786

**Published:** 2025-02-20

**Authors:** Clara L. Marx, Laura M. König

**Affiliations:** ^1^ Faculty of Life Sciences: Food, Nutrition and Health University of Bayreuth Bayreuth Germany; ^2^ Faculty of Psychology University of Vienna Vienna Austria

**Keywords:** learning, media psychology, nutrition education, social media, YouTube

## Abstract

**Objectives:**

Online videos are becoming increasingly popular for obtaining nutrition‐related information. Learning theories suggest that videos may differ in their effectiveness of conveying knowledge depending on the correspondence between audio and visual content. We thus tested whether two popular video formats, i.e. *talking‐head* and *animated video*s, differed regarding knowledge transfer effectiveness and their ability to stimulate content sharing.

**Design:**

2 video format x 3 topic between‐subjects experiment.

**Methods:**

A total of 358 participants who were representative for the German population regarding age, gender and level of education were randomly assigned to viewing one video format about one of three nutrition‐related topics. Afterwards, they rated the video, indicated willingness to share the information with others and answered a set of quiz questions about all three topics to assess knowledge.

**Results:**

Videos did not differ in their evaluation (*F[*1, 352] = 0.16, *p* = .898), knowledge transfer (*F*[2, 352] = 0.10, *p* = .749) or content sharing (*F*[1, 352] = 0.12, *p* = .727). However, participants received a better knowledge score for the video topic they watched a video about than for the other two topics (*F*[4, 704] = 50.00, *p* < .001, partial η2 = .22).

**Conclusions:**

Therefore, both formats can be considered equally effective for use in science communication.

**Statement of contribution**

**What is already known on the subject?**

- Social media is an important channel for lay people to retrieve information about healthy diets and nutrition.
- According to learning theories and empirical evidence, videos are effective tools for science communication since they combine auditory and visual channels.

**What does the study add?**

- No significant differences between video formats in knowledge and willingness to share indicate that both talking‐head and animated videos are effective for science communication.
- Further characteristics (e.g. video length, narrative structure) require attention.

## INTRODUCTION

The prevalence of diet‐related diseases like type II diabetes mellitus is increasing globally (Ong et al., 2023). There is thus a need for effective public health strategies to combat this trend, such as communication about the findings of nutritional science and medical research. Due to the increased popularity of social media, nutrition communication often takes place online in various contexts (Godemann & Bartelmeß, 2021). Several actors are involved in online nutrition communication, including lay people, brands, and influencers; yet they often spread inaccurate information (Denniss et al., 2023) which may have serious consequences for health and well‐being of the population (Sina et al., 2022). To counteract the spread of misinformation, nutrition professionals as well as science communicators need to engage in the discussion and use effective communication strategies.

Social media is used by the majority of the world's population (Dixon, 2024 April 10). It is not only an important pastime activity, but also used by many individuals to obtain information about science (Mede et al., 2024). Effective science communication on social media requires a thorough understanding of the cognitive mechanisms of learning (Mayer, 2014), if the main aim of the communication is to convey knowledge. In the field of instructional psychology, various theories explain the interactions between cognitive processes and the design of learning environments. Sweller's Cognitive Load Theory (CLT) (1994) states that human cognitive capacity is limited. According to CLT, every task has an inherent internal complexity and an external complexity determined by task design. Therefore, tasks with high internal complexity require reducing external complexity to prevent cognitive overload and enable effective learning (Sweller, 1994). Mayer and Moreno's Cognitive Theory of Multimedia Learning (CTML) (1998) extends this theory to multimedia. CTML emphasizes active cognitive processing of information and underlines the importance of selecting, organizing, and integrating information in working memory, which demands cognitive capacity (Mayer, 2014).

Both CLT and CTML highlight that cognitive overload hinders learning. Even though long‐term memory may have unlimited capacity, every piece of information must initially pass through the limited working memory. If cognitive capacities are overloaded, the processes of selecting, organizing, and integrating information into long‐term memory can no longer be effectively executed, leading to less information being retained. Cognitive overload can particularly occur with audiovisual media, as they simultaneously address both audio and visual channels (“dual‐channel assumption”) (Boy et al., 2020; Bucher et al., 2022; Mayer & Moreno, 1998).

According to CLT and CTML, educational media, including media created for science communication, need to be carefully crafted to avoid cognitive overload and so promote learning. In media using multiple channels, such as videos, this could be achieved by aligning audio and visual channels. For instance, animated videos allow to visualize numbers and verbatim reproductions and so aligns the spoken text with the visuals (Boy et al., 2020). However, if a video mainly shows a person standing in front of the camera and talking (“talking‐head video”), which is the most popular format for science communication videos (Beautemps & Bresges, 2021), this alignment of channels is often lacking. Consequently, talking‐head videos should be less effective than animated videos in conveying knowledge. On the one hand, Boy et al. (2020) demonstrated that animated videos indeed convey knowledge more effectively than talking‐head videos. It can be assumed that animated videos direct the viewer's gaze more effectively, potentially because there is no moderator to distract attention. Another study by Wiseman (2015) also confirmed the superiority of animated videos in knowledge transfer, suggesting that animated videos might be the preferred format for effective science communication. On the other hand, in a comparison of PowerPoint and animated whiteboard videos, Draijer (2020) found that both formats were equally effective in conveying knowledge, although respondents were more satisfied with the animated whiteboard videos. Also, a study by Sondermann and Merkt (2023), in which the authors compared different types of narrated slides, showed no significant influence of talking‐heads. One reason for the inconclusive findings might be methodological shortcomings: The videos compared in previous studies were either different in content (Boy et al., 2020), very short (Wiseman, 2015), or compared animated videos with a format other than talking‐head videos (Draijer, 2020) and vice versa (Sondermann & Merkt, 2023). To be able to draw firm conclusions regarding whether animated videos are indeed superior to talking‐head videos, more realistic stimulus material presenting the same content is required.

Despite playing a crucial role in memory consolidation, cognitive load might not be the only key determinant of learning success. Moreno's Cognitive‐Affective Theory of Learning with Media (CATLM) (2006) proposes to add affective aspects, stating that individual motivational factors affect cognitive engagement and learning processes. Schneider et al. (2022) further developed CATLM into the Cognitive‐Affective‐Social Theory of Learning in digital Environments (CASTLE), which highlights the influence of social signals that can arise in digital media use on learning. It could be assumed that talking‐head videos emit stronger social signals than animated videos because a presenter is visible. This visibility, in line with the principles of CASTLE, could lead to greater learning success and thus could also be a reason for the diverging study results.

To overcome methodological shortcomings of prior studies and to consolidate the inconclusive findings and assumptions based on different learning theories, the present study aimed to compare animated and talking‐head videos that were created based on the same audio file. The stimuli required for this test were adapted from Weiß and König (2023), who previously examined the effectiveness of communicating nutrition‐related information using texts, podcasts, and videos with identical content and moderation. They found no significant differences in knowledge transfer effectiveness among the media formats, so questioning the signalling principle postulated in the CTML. This principle postulates that learners achieve greater success when they can see the information rather than just hear it, allowing for better concentration (Mayer, 2014). A potential reason for this could have been the use of talking‐head instead of animated videos; the present study examines this assumption. Specifically, it tests the following research questions (RQ; see Table 1 for hypotheses):

**TABLE 1:** Overview of research questions and hypotheses and links to theories.

RQ no.	Hypothesis	Theory
1	H1.1: Animated videos will be more effective in conveying nutrition‐related information than talking‐head videos.	CTML
	H1.2: Animated videos will be more effective in conveying factual knowledge in form of numbers than talking‐head videos.	CTML
	H1.3: Animated videos will be more effective in conveying factual knowledge presented verbatim in the video than talking‐head videos.	CTML
	H1.4: Talking‐head videos will be more effective in conveying factual knowledge that must be inferred from the context of the video than animated videos.	CASTLE
2	H2.1: Animated videos will be more effective in conveying nutrition‐related information than talking‐head videos.	CTML
	H2.2: Animated videos are more effective at conveying factual knowledge in numerical form.	CTML
	H2.3: Animated videos are more effective at conveying factual knowledge presented verbatim in the video.	CTML
	H2.4: Talking‐head videos are more effective at conveying factual knowledge that requires inference from the video's context.	CASTLE

RQ1: Do animated videos and talking‐head videos differ in their effectiveness to convey nutrition‐related information, as indicated by a difference in knowledge scores between experimental conditions?

RQ2: Do animated videos and talking‐head videos differ in their effectiveness to convey nutrition‐related information, as indicated by differences in knowledge about topics addressed vs. not addressed in the video?

Importantly, knowledge gain is only one of many science communication objectives (c.f. Ziegler & Fischer, 2020). Especially in the context of social media, which allows users to interact with and share the content they consume, willingness to share the content is crucial. This would also contribute to spreading correct instead of false information. Indeed, Wiseman (2015) reported that animated videos were shared more often than talking‐head videos, potentially, because they were rated as more interesting, entertaining and informative. Accordingly, this study also investigated the following exploratory research question:

RQ3: Do animated videos and talking‐head videos differ in their effectiveness to stimulate participation in the form of sharing the videos?

## METHODS

The study was preregistered on aspredicted.org prior to data collection: https://aspredicted.org/fcvg‐g5n9.pdf. Materials and data are available on the project's Open Science Framework page (https://osf.io/4u6bc/). The study was approved by the University of Bayreuth ethics committee.

### Sample

According to Boy et al. (2020), a small to medium effect was expected for differences between video formats (Cohen's *f* = 0.15); G*Power (Faul et al., 2009) yielded a sample of *N* = 351 for 80% power and *α* = .05 for a 2 (medium) × 3 (topic) between‐subjects ANOVA. Accordingly, data was collected until there were 59 participants per group, yielding a total of *N* = 354 participants. A sample representative for the German population in terms of gender, age and education was recruited through a panel provider. Those under 18 were automatically excluded and replaced during data collection. Participants who failed two quality checks were also excluded and replaced.

After the initial data collection was completed, we noticed that participants who were supposed to view the animated videos for the topics sugar and nudging indeed viewed the respective talking‐head videos, which rendered *N* = 119 participants of the original sample invalid. Their data was replaced with a newly recruited sample (see https://osf.io/4nqpg for the amendment to the preregistration).

### Material and measures

The materials and measures utilized in this study were adapted from Weiß and König (2023).

#### Talking‐head videos

In their pilot study, Weiß and König (2023) asked 24 participants to indicate their interest in seven distinct food and nutrition‐related topics, while also evaluating their knowledge on these subjects. From this assessment, three topics that garnered the highest interest and demonstrated comparable levels of knowledge were selected: the impact of nutrition on climate, the role of sugar in foods, and the influence of nudges on eating behaviour. To ensure comparability among the videos, the authors crafted scripts of approximately equal length (9 minutes of content) for each of the three topics, which were then used to produce the talking‐head videos. First drafts were written by a student of food and health sciences, and then iteratively revised by academics with experience in science communication research and practice and professional science communicators and journalists. In these videos, a Caucasian woman in her twenties spoke directly into the camera and moderated all three videos, following standard practice in many popular science communication YouTube channels. A few static images and graphs were blended in and displayed in full screen. To create the appearance of a YouTube channel, a logo featuring the phrase ‘9 minutes of nutrition sciences’ was designed and shown in the beginning of the video. The links to the original talking‐head videos (in German) can be found in Table [Link], [Link]; the video scripts (German original and English translation) are available from https://osf.io/nmh8u/.

#### Animated videos

**FIGURE 1:**
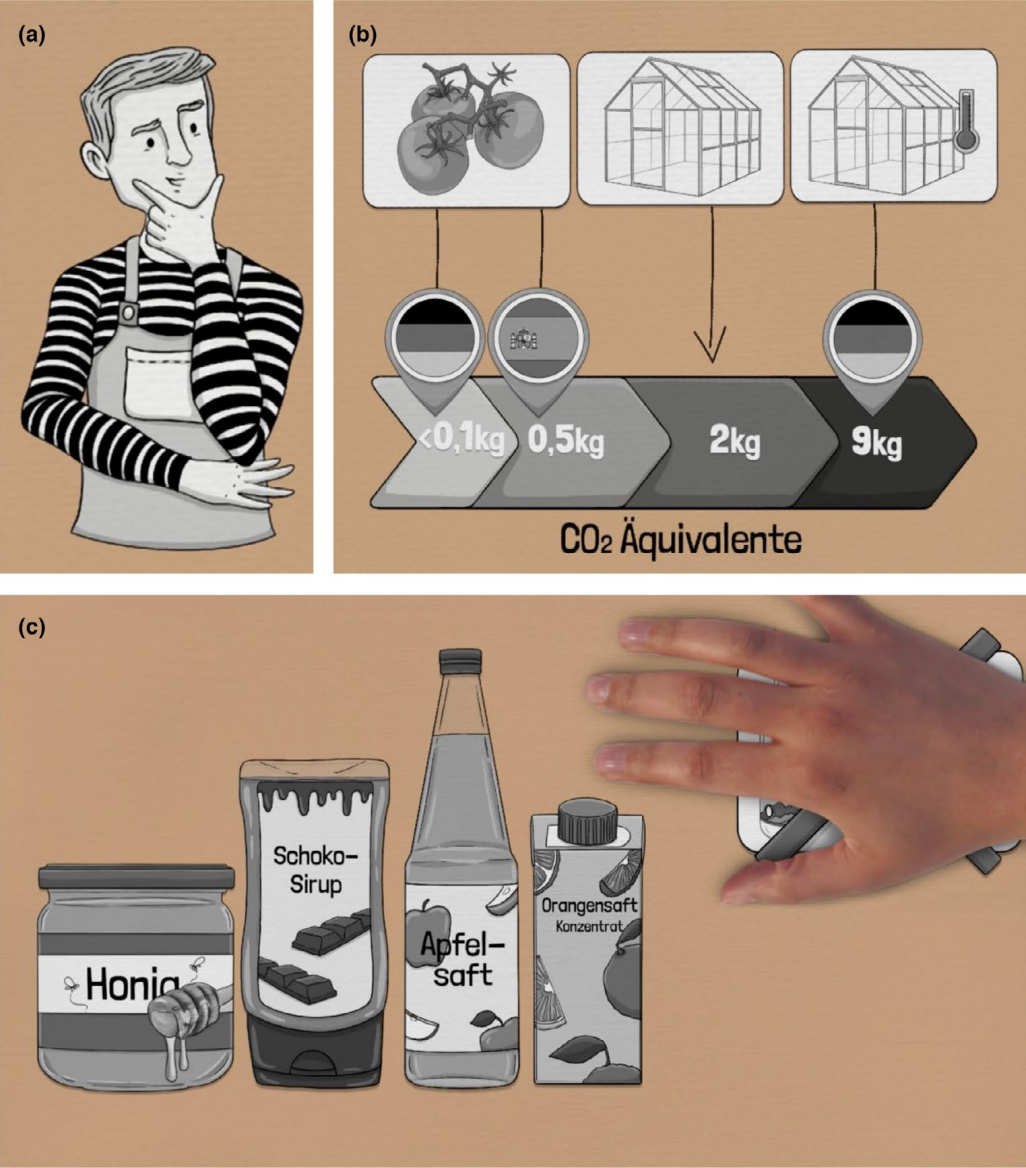
Excerpt from the animated video on the topic of nudging (a), the topic of climate (b) and the topic of sugar (c).

In addition to the talking‐head videos already used in Weiß and König (2023), we created animated videos for this study by drawing 2D illustrations with Procreate® software (Savage Interactive Pty Ltd.), which we then transferred to Videoscribe software (Sparkol). To ensure high comparability of the video formats, we used the same audio track for the animated videos as for the talking‐head videos. The animated videos were designed according to recommendations by Lowe and Schnotz (2008) to avoid cognitive overload (Bucher et al., 2022; Mayer, 2014). Specifically, we aligned the illustrations closely with the spoken text and avoided purely decorative images that could distract viewers (Franker, 2020; König et al., 2025). Furthermore, we used animations so that elements addressed in the audio track were emphasized, for example by zooming in, to guide viewers' attention and to ensure that spoken text and corresponding animation are presented simultaneously (Bucher et al., 2022; Mayer, 2014). Finally, we used warm, saturated colours and anthropomorphic figures (see Figure 1) to evoke positive emotions in viewers (Plass et al., 2014), which can influence learning success (Heidig et al., 2015; Wong & Adesope, 2021). To keep cognitive load low and to maintain uniformity across the three animated videos, we designed individual elements in neutral grey tones. The links to the animated videos (in German) can be found in the Data S1.

#### Questionnaire

The translated questionnaire can be found in the Data S1. The original German version is available from the study's OSF Page (https://osf.io/4u6bc/).

##### Interest and attitudes

Participants indicated their interest in nutrition, health, sustainability, science, and research on a 5‐point Likert scale, with ratings from 1 = *not at all* to 5 = *very* for nutrition, health, and sustainability and from 1 = *very low* to 5 = *very large* for science and research. Trust in science and researchers, particularly in universities, research institutions, industries, and businesses was assessed with three items from (Wissenschaft im Dialog & Kantar Emnid, 2019).

##### Media use

We used a six‐point Likert scale ranging from 1 = *never* to 6 = *(almost) daily* to assess the frequency of viewing animated and talking‐head videos about nutrition‐related topics.

##### Evaluation of the video

After viewing the video, participants were asked to evaluate the video and its content. The items for general or content‐related evaluation were sourced from Weiß and König (2023) and were presented as six‐point semantic differentials (e.g. *bad* vs. *good*, *not at all interesting* vs. *very interesting*). For content‐related evaluation, three item pairs were used, such as *very untrustworthy* vs. *very trustworthy*.

##### Intention to share

Participants were asked if they would share the information in a personal conversation and/or share it on social media on a 7‐point Likert scale ranging from 1 = *no certainly not* to 7 = *yes definitely* (c.f., Giese et al., 2021; Kotz et al., 2023).

##### Knowledge

We assessed participants' knowledge regarding all three topics, irrespective of the video they watched. We added three questions to the original questionnaire used in Weiß and König (2023) so that knowledge about each topic was assessed with eight questions. These included inquiries related to numbers mentioned in the video, questions with answers directly mentioned in the video, and questions that required deduction from the context. To add variety and prevent boredom, some questions were designed as multiple‐choice or ranking format.

### Design and procedure

**FIGURE 2:**
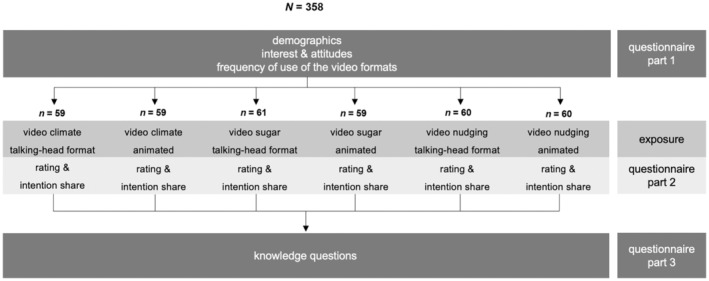
Procedure of the study and number of participants per experimental group.

A randomized experimental study was executed with a 2 (Video formats: talking‐head or animated) × 3 (Topic: climate, sugar, or nudging) between‐subjects design (Figure 2); the dependent variable knowledge was assessed within‐subjects.

The survey was administered online via Tivian Unipark. Participants first provided informed consent by ticking a box. We then assessed demographic information, interests and attitudes on nutrition, sustainability, and scientific research, and video consumption. Participants were then randomly assigned by the questionnaire tool to one of the six conditions and asked to watch a video. To achieve equal group sizes, participants randomized to a condition that already achieved the target sample size were screened out. After viewing, they were asked to evaluate the video and express their intention to share its information in personal conversations or on social media. Finally, they completed a quiz to assess their knowledge about all three topics.

### Data analysis

We used IBM SPSS Statistics (29.0.1.0 (171)) and JASP 0.18.3.0 (JASP Team, 2024) to analyse the data. There were no missing values as responses were mandatory.

#### Preprocessing

##### Evaluation of the videos

Participants evaluated the videos overall and their specific content using various items. We first calculated Cronbach's alpha to verify if the items could be combined into a single construct (see Table 2). As all values were above 0.7 (Gliem & Gliem, 2003), the items for video evaluation were combined into *general rating* and *content rating* constructs.

**TABLE 2:** Cronbach's α and Pearson correlation for the items used to evaluate the videos.

Topic	Format	Cronbach's α general rating	Cronbach's α content rating	*r*	*p*
Climate	Talking‐head	0.96	0.96	.51	<.001
	Animated	0.97	0.93	.71	<.001
Sugar	Talking‐head	0.94	0.85	.64	<.001
	Animated	0.96	0.98	.58	<.001
Nudging	Talking‐head	0.97	0.95	.63	<.001
	Animated	0.96	0.95	.67	<.001

##### Intention to share video content

To determine if the two items used to measure intention can be combined into one construct, we calculated Pearson correlations (Sondermann et al., 2024). All correlations indicated large effects (Cohen, 1992), so we consolidated the two items into the *Intention to Share* construct.

##### Knowledge score

A measure for knowledge transfer was calculated following the procedure outlined in Weiß and König (2023). For each question, a new variable was created where the correct answer was coded as 1 and all incorrect answers as 0. Multiple‐choice or ranking questions were only deemed correct if fully answered correctly. The Knowledge Score was then calculated as the percentage of correct answers out of all answered questions. In addition, as we also hypothesized differences regarding different forms of knowledge, we calculated separate scores for questions asking for *numbers*, questions asking to reproduce information *verbatim*, and questions asking about information that has to *be inferred from the context*.

##### Video duration

To ensure participants watched the assigned video, the “Next page” button only appeared after 5 minutes into the video intervention, the maximum possible duration in the survey tool. The actual duration spent on the page that displayed the video was calculated based on the relative timestamps provided by the survey tool. Participants were categorized based on the time they spent on the video page. For participants who stayed below 9 or over 20 min (i.e. approximately twice the length of the videos to account for potentially watching them twice), we have to assume incomplete or improper watching.

#### Preregistered statistical analyses

Statistical analyses were preregistered prior to the start of data collection (https://aspredicted.org/P29_B8V). To assess differences in the effectiveness to convey nutrition‐related information of animated videos and talking‐head videos, as indicated by a difference in knowledge scores between experimental conditions (RQ1), we conducted 2 *Format* x 3 *Topic* between‐subjects ANOVAs, using the overall and question category‐specific knowledge scores as the dependent variable. To evaluate RQ1, the main effect of format is relevant.

To assess differences in the effectiveness to convey nutrition‐related information of animated videos and talking‐head videos, as indicated by differences in knowledge about topics addressed vs. not addressed in the video (RQ2), we conducted a mixed ANOVA, using the group allocation as independent variable (between‐subjects) and the knowledge score as the dependent variable (within‐subjects) (c.f. Weiß & König, 2023). To evaluate RQ2, the interaction effect format x topic x knowledge score is relevant.

To assess whether animated videos and talking‐head videos differ in their effectiveness to stimulate participation in the form of sharing the videos (RQ3), we conducted a between‐subjects ANOVA, using topic and video format as independent variables.

#### Additional analyses

In addition to the preregistered analyses, the following analyses were conducted.

##### Manipulation check

We explicitly report the topic x knowledge interaction term in the ANOVA conducted to address research question 2 as a manipulation check to confirm that the videos generally increased knowledge.

##### Bayesian analysis to follow up null effects

Following suggestions during peer review, we followed up the null effects obtained for the confirmatory research questions (RQ1, RQ2), we repeated the tests of the models using the respective Bayesian ANOVA in JASP 0.18.3.0 (JASP Team, 2024). Due to a lack of informative priors, we used default priors. Models were compared to the null model, but due to the large number of effects, we only report the effects across models. Since the aim of this analysis was to generate information about the null effects, we report BF_01_, i.e. the Bayes factor for H0 over H1. The corresponding JASP project is available from the study's OSF page (https://osf.io/4u6bc/).

##### Influence of video evaluation

To explore whether participants who rated the videos higher also achieved higher knowledge scores, we calculated a Pearson correlation.

##### Influence of watch time

To explore whether responses differed based on having watched the video complete, we ran all analyses again with the additional between‐subjects factor *Completion* (yes/no).

## RESULTS

### Sample characteristics

A total of *N* = 358 people participated in the study, which included 50% men, 49.7% women, and 0.3% diverse individuals. Participants' ages ranged from 18 to 78 years with an average age of 46.5 years (*SD* = 15.7). Among them, 30.7% had a high school diploma, 24% completed primary school, and 22.6% had a middle school education. More than half of the participants (57.8%) were employed, while 21.2% were retired. Additionally, 7.5% were in training or studying, 6.7% were unemployed, and the same number were homemakers. As per the total sample, 2.5% of the participants were studying a subject related to nutrition, such as medicine. No participant held a job related to nutrition. There were no statistically significant differences between experimental conditions with regards to the participants gender, age, education, and employment status (see Table S2).

Participants showed interest in the topics of nutrition (*M* = 3.80, *SD* = 1.09), health (*M* = 3.99, *SD* = 1.07), and sustainability (*M* = 3.56, *SD* = 1.20). They also had rather high trust in science and research (*M* = 3.66, *SD* = 1.02) and scientists in both academia (*M* = 3.61, *SD* = 1.07) and industry (*M* = 3.23, *SD* = 1.04), with 63.5%, 59.2% and 40.1% indicating to rather or fully trust them, respectively.

Most participants reported to never or only rarely watching talking‐head or animated videos. In relation to nutrition topics, even more participants reported never or rarely watching videos in these formats (see Data S1 for details).

### Manipulation check: effectiveness of videos to convey knowledge

**FIGURE 3:**
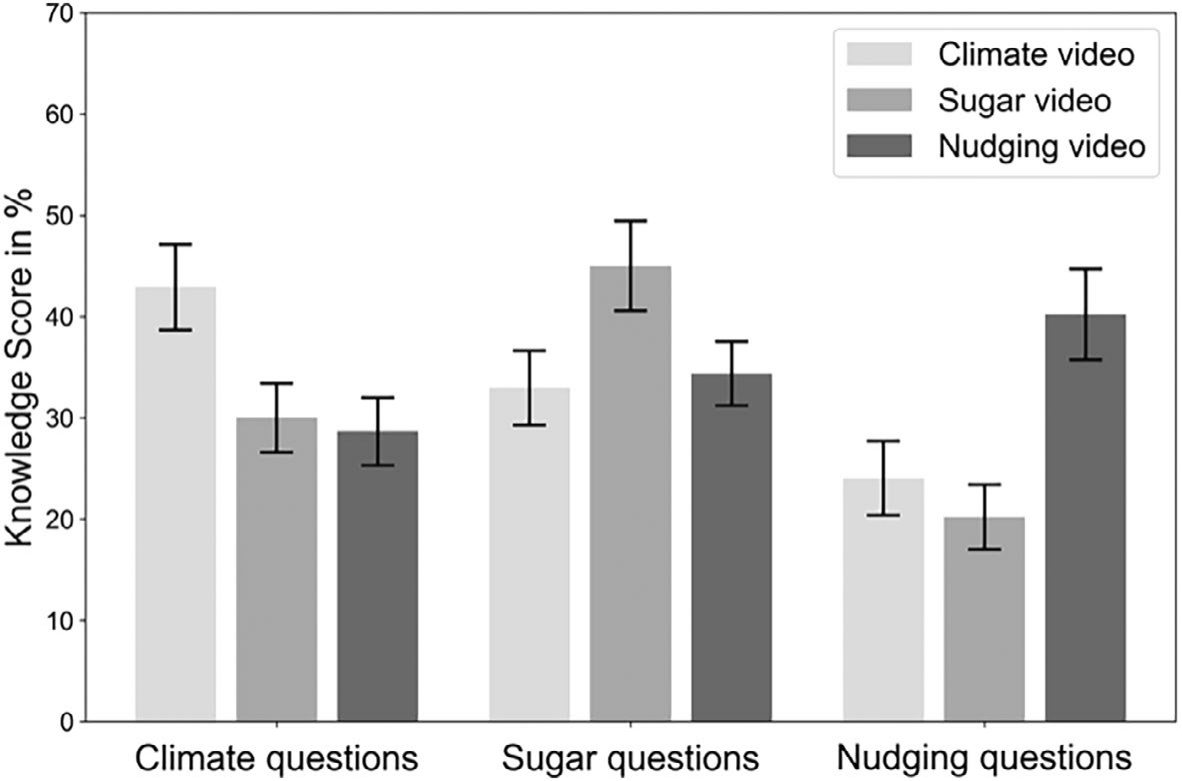
Knowledge Scores in relation to the topic of the questions. Error bars indicate the standard errors.

A mixed ANOVA was conducted, using video format and topic as between‐subjects factors and the knowledge score per topic as the within‐subjects factor. To test whether the videos were generally effective in increasing participants' knowledge, we focused on the topic x knowledge interaction as a manipulation check. Indeed, the interaction was statistically significant, indicating that participants knew more about the topic that they received a video about, *F* (4, 704) = 50.00, *p* < .001, partial η^2^ = 0.22 (see Figure 3).

### Testing for differences in the effectiveness of knowledge transfer

According to Hypothesis H1.1, animated videos should outperform talking‐head videos in knowledge transfer. However, the ANOVA yielded no significant main effect for video format on the overall knowledge score, *F* (2, 352) = 0.10, *p* = .749, partial η^2^ = 0.00. Similarly, no significant main effects emerged for the question category‐specific scores (numbers: *F* (2, 352) = 0.83, *p* = .364, partial η^2^ = 0.00; verbatim: *F* (2,352) = 0.36, *p* = .547, partial η^2^ = 0.00; infer: *F* (2, 352) = 0.31, *p* = .576, partial η^2^ = 0.00). Null results were confirmed by the corresponding Bayesian between‐subjects ANOVAs which provided substantial to strong evidence for removing the main effect for the video format from the models (BFs_excl_ ranging from 8.30 to 11.71; see also Table S3). Therefore, hypotheses H1.1–1.4 could not be accepted.

### Differences based on the video topic

In a next step and following the analysis procedure in Weiß and König (2023), potential differences in the effectiveness to convey knowledge by topic were tested in a mixed ANOVA, using video format and topic as between‐subjects factors and the knowledge score per topic as the within‐subjects factor. There was no significant main effect of topic, indicating that across conditions, participants had similar knowledge about the topics, *F* (2, 352) = 0.78, *p* = .461, partial η^2^ = 0.00. To evaluate H2.1, we specifically tested for a three‐way interaction between video format, topic, and knowledge score; this interaction was not statistically significant, *F* (4, 704) = 1.15, *p* = .333, partial η^2^ = 0.01. The results were supported by a Bayesian mixed ANOVA, which yielded decisive evidence for the three‐way interaction term to be excluded from the model (c.f. H2.1; BF_excl_ = 380.48; see also Table S3). H2.1 thus could not be accepted.

We repeated this analysis for the question category‐specific knowledge scores. For questions referring to numbers, no significant main effect of the topic was observed, *F* (2, 352) = 0.76, *p* = .467, partial η^2^ = 0.00. The three‐way interaction between video format, topic, and knowledge score was also not significant, *F* (4, 704) = 0.71, *p* = .589, partial η^2^ = 0.00. This result was supported by a Bayesian mixed ANOVA, which yielded decisive evidence for the three‐way interaction term to be excluded from the model (c.f. H2.2; BF_excl_ = 2398.61; see also Table S3).

For questions whose content was mentioned verbatim in the video, there was also no significant main effect of the topic, *F* (2, 352) = 0.95, *p* = .389, partial η^2^ = 0.01. The three‐way interaction between video format, topic, and knowledge score was again not significant, *F* (4, 704) = 1.08, *p* = .364, partial η^2^ = 0.01. Results were confirmed by a Bayesian mixed ANOVA, which yielded decisive evidence for the three‐way interaction term to be excluded from the model (c.f. H2.3; BF_excl_ = 1519.82; see also Table S3).

Regarding the questions for which a response needed to be inferred from the context, no significant main effect of topic emerged, *F* (2, 349) = 2.20, *p* = .112, partial η^2^ = 0.01. The three‐way interaction between video format, topic, and knowledge score was also not significant, *F* (3.87, 675.04) = 0.64, *p* = .630, partial η^2^ = 0.00. A Bayesian mixed ANOVA confirmed these results with decisive evidence for the three‐way interaction term to be excluded from the model (c.f. H2.3; BF_excl_ = 6151.13; see also Table S3). In sum, H2.2–H2.4 could also not be accepted.

### Testing for differences in the intention to share

On a seven‐point Likert scale participants reported their interest in sharing the videos with *M* = 3.42 (*SD* = 1.83); on average they were thus neither in favour nor against sharing. When considering the two items that inquired about intention individually, the intention to discuss the content in personal conversations (*M* = 3.91, *SD* = 2.04) was higher than the intention to share the content on social media (*M* = 2.94, *SD* = 2.02; *t* (357) = 10.51, *p* < .001). The video formats did not differ in intention to share, *F* (1, 352) = 0.12, *p* = .727, partial η^2^ = 0.00.

### Influence of video evaluation

The two video formats did not differ in terms of overall evaluation (talking‐head: *M* = 4.22, *SD =* 1.25; animated: *M* = 4.24, *SD* = 1.35; *F* (1, 352) = 0.16, *p* = .898, partial η^2^ = 0.00) or evaluation of the content (talking‐head: *M* = 4.56, *SD* = 1.25; animated: *M* = 4.52, *SD* = 1.27; *F* (1, 352) = 0.07, *p* = .789, partial η^2^ = 0.00). When split by video topics, the sugar video received the highest ratings, with a general score of *M* = 4.40 (*SD* = 1.17) and a content score of *M* = 4.74 (*SD* = 1.08). The nudging and climate videos scored slightly lower, but the difference was not significant (general: *F* (2, 352) = 1.83, *p* = .161, partial η^2^ = 0.01; content‐wise: *F* (2, 352) = 2.37, *p* = .095, partial η^2^ = 0.01).

A Pearson correlation showed a statistically significant increase in Knowledge Score when the video received better general ratings (*r* = .35, *p* < .001) and more favourable ratings in terms of content (*r = .36*, *p <* .001).

### Influence of watch time

Values exceeding 20 min of watch time were summarized as “20 min or above”, as this duration is more than twice the length of the videos. Out of 358 participants, 6% (*n* = 22) spent over 20 min on the page containing the video. Another 155 participants stopped the video before reaching 9 min, out of which 103 stopped within 5–6 min, i.e. immediately after the “next page” button appeared. A similar number (*n* = 142) spent between nine and 11 min with the videos, equivalent to the videos' length. Viewing times did not differ based on video topic (*F* (2, 352) = 0.99, *p* = .374) or format (*F* (1, 352) = 1.03, *p* = .311) according to a between‐subjects ANOVA; also the interaction was not statistically significant (*F* (12, Obj 352) = 0.93, *p* = .392).

**FIGURE 4:**
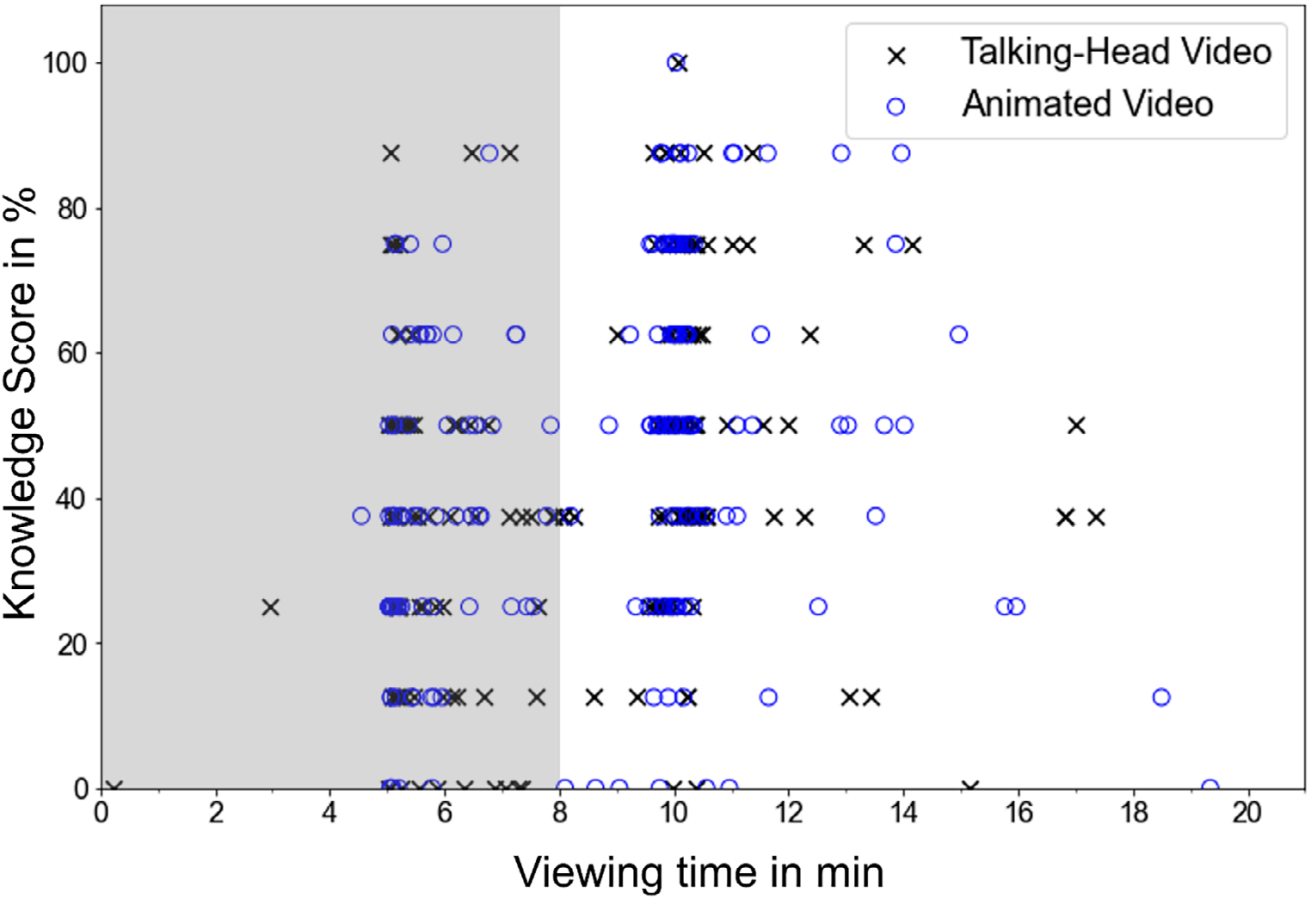
Time participants spent watching the video in relation to the achieved knowledge score. Participants with watch times below nine or over 20 min (Group 1) are highlighted through grey background.

Due to the differences in viewing times, we conducted an additional exploratory analysis to ascertain whether a non‐significant result in the analysis of the total group was due to insufficient video duration. Figure 4 provides an overview of the varying durations of the subjects' viewing time.

The group that lingered with the video for under nine or over 20 min (Group 1) comprised 177 people. The group with a duration between nine and 20 min (Group 2) consisted of 181 people. On average, participants in Group 1 achieved a Knowledge Score of *M* = 34.81, *SD* = 22.5, while those in Group 2 scored an average of *M* = 50.4, *SD* = 23.9. This difference in the average Knowledge Scores was significant (*t* (356) = 6.36, *p* < .001).

Due to the potential effect of having watched the video completely and attentively, we repeated the analyses with only the participants who watched for between 9 and 20 min. Also, this subsample did not show a significant main effect of *Video Format* on the *Knowledge Score*, *F* (1, 175) = 0.00, *p* = .98, partial η^2^ = 0.00 (H1.1). Results were similar when the Knowledge Score was separated into individual question categories (H1.2–H1.4).

As in the full sample, the subsample analysis also yielded a significant interaction effect between the question topic and the topic of the viewed video, *F* (4, 350) = 42.44, *p* < .001, partial η^2^ = 0.33. Results were again similar when the Knowledge Score was separated into individual question categories (H2.2–H2.4).

Finally, we also compared the two subsamples regarding intention to share video content. Group 2, with an average of *M* = 3.54 (*SD* = 1.8), indicated a slightly stronger intention to share content than Group 1, with an average of *M* = 3.3 (*SD* = 1.87). However, this difference was not significant (*t* (356) = 1.25, *p* = .106). The main effect of *video format* on the intention to share the video was again not statistically significant in the subsample that watched the video completely, *F* (1, 175) = 0.05, *p* = .818, partial η^2^ = 0.00.

## DISCUSSION

This study used a representative German sample (Statistisches Bundesamt, 2022) to compare animated and talking‐head videos in the effectiveness in conveying nutrition‐related knowledge and the ability to promote content sharing, which are two main goals of science communication on social media (Lungu et al., 2021; Ziegler & Fischer, 2020). Participants knew more about the topic that they were exposed to in the video compared to the other topics, as previously suggested by Weiß and König (2023), but the two video formats performed comparably well. Notably, the subjects who rated the videos better also performed better in the knowledge test, pointing towards the important role of individual preferences in communication style and content, which needs to be carefully reflected upon and addressed by the communicator (Humm & Schrögel, 2020).

### Influence of video formats on knowledge transfer

The lack of differences between the two video formats could be due to different advantages and disadvantages of the video formats cancelling each other out. On the one hand, the animated video offers clear visual guidance and low cognitive load which should promote learning according to CLT and CTML (Bucher et al., 2022; Mayer, 2014; Sweller, 1994). On the other hand, the talking‐head video delivers stronger social signals, due to the visible moderating person and potentially enhances the learning effect due to the activation of social schemata as postulated in CASTLE (Schneider et al., 2022). Similar effects were reported in other studies on the effect of talking‐heads on knowledge transfer via videos (Alemdag, 2022; Henderson & Schroeder, 2021; Polat, 2023; Sondermann et al., 2024).

Alemdag (2022) for example notes that while the presence of an instructor did not have statistically significant effects on learning and social presence, it did significantly enhance cognitive load and motivation.

Initially, we hypothesized that individuals who watched animated videos would perform better on numerical and verbatim questions due to the direct alignment of auditory and visual content presentation. We also hypothesized that those who viewed the talking‐head videos would perform better in inferential questions because of the stronger social cues (Sondermann et al., 2024). However, the lack of differences between video formats regarding the overall knowledge score was mirrored when comparing different forms of knowledge questions.

The lack of significant differences between video formats in this study contradicts the findings of Boy et al. (2020) and Wiseman (2015). The results reported in Boy et al. (2020) suggested a medium effect size, which we also used to calculate the sample size for the present study. However, Boy et al. (2020) used different videos for the different conditions, so their results could also be attributed to other differences between the stimuli. In the present study, we deliberately used the same audio file – and thus narrative structure – in both videos to ensure that both video format groups received the same information. Indeed, prior research highlights the importance of narrative structure for knowledge transfer (Boy et al., 2020; Bucher et al., 2022; Hecker et al., 2018; Schneider et al., 2023). While the present study cannot rule out small differences in effectiveness to convey knowledge between talking heard and animated videos, comparing the results to prior studies suggest that these effects might be masked by the stronger influence of the narrative structure.

Wiseman (2015) surveyed over 2000 individuals and thus this study was powered to detect even very small effects. Unfortunately, based on the available information on this study, it is not possible to calculate the effect size. Based on the reported 15% increase in remembered information, however, it is to be assumed that effect sizes were similar, given that the animation video groups achieved overall knowledge scores that were between 11% lower and 22.5% higher than the talking‐head video group. However, the videos used in Wiseman (2015) were only about a minute long. Similarly, Manasrah et al. (2021) also showed that short videos had a significantly positive impact on online lecture grades. In this study, optimal video length was determined to be 6–10 min (Manasrah et al., 2021), which is consistent with the videos in the present study. However, nearly half (49.44%) of the subjects did not watch the videos until the end, suggesting that the videos may have been too long. Indeed, according to cognitive learning theories like CLT and CTML, longer videos might overload the limited capacity of working memory. This aligns with Draijer's (2020) study of 15‐minute videos, which found no differences in knowledge transfer effectiveness across video formats, similar to the present study, where we used 9 min videos. Future research might explore the use of 6–7 min long videos when comparing formats, and test whether the video length moderates the impact of video format on watching the video to the end and learning success.

### Influence of video formats on the intention to share the content

The video format did not affect participants' intent to share the content; on average, participants were neither interested in sharing the content in a personal conversation or via social media nor particularly against it. Since evaluations of the videos were generally positive, it is unlikely that this lack of sharing intention was due to lack of interest. The lack of intention might rather be due to them rarely consuming this type of video in general. Indeed, educational content is rarely shared, potentially because it is seen as more effortful or less interesting to watch compared to purely entertaining content (Shortt et al., 2022; Yang & Wang, 2015). Several features of a video may contribute to it being shared; for science videos specifically, they might be more frequently shared if viewers find them useful and if the provided information is self‐enhancing (Milkman & Berger, 2014). Also emotional (science) news are more likely shared, although research is inconsistent as to whether this only applies to negative, or also positive valence (Milkman & Berger, 2014; Watson et al., 2024). The relatively neutral presentation of information in the videos used in the present study therefore might have contributed to the intention to share being relatively low.

### Influence of the evaluation

Participants who rated the videos better also performed better in the knowledge test; the correlation between knowledge score and evaluation represents a medium effect (Cohen, 1992). This finding can be explained by the CASTLE model (Schneider et al., 2022). According to their *Interaction Hypothesis*, social processes and motivational processes influence each other, and both enhance learning success (Mayer, 2014; Mayer & Moreno, 1998; Schneider et al., 2022; Sweller, 1994). A favourable video evaluation can indicate positive emotions towards the videos and, consequently, increased attention, which should lead to better performance. On the other hand, participants who rated the video poorly might be dissatisfied with the task and not answer questions as carefully (Verhallen & Pieters, 1984). They may perceive it as unrewarding to invest cognitive capacity in something they did not enjoy. However, as the two video formats received similar average ratings, it is unlikely that the ratings influenced the overall findings of the study. Furthermore, although videos were positively received by the audience, they did not receive perfect ratings. Indeed, science communication content often does not make use of more creative methods and art styles (Shortt et al., 2021). More creative videos may be seen as more engaging, yet many recipients of science communication might expect a relatively neutral tone based on their stereotypes of scientists (König et al., 2025); the effect of creative presentation styles on the effectiveness of science communication remains to be tested.

Importantly, almost half of participants did not finish watching the video, which can be seen as an additional indicator of their interest, which may seem limited. Although this could also be a bias of the sample, which was recruited via a panel provider and therefore mostly interested in completing the survey quickly to receive their reward, this also highlights the struggle of science communicators to capture the attention of their audience. Several factors may influence interest, including statements of scientific consensus (König et al., 2025) and relevance to the recipient's daily life (Humm & Schrögel, 2020); especially the latter varies between persons and points towards the crucial role of tailored communication.

### Strengths and limitations

The sample was representative for the German population in terms of age distribution, gender ratio and level of education; this also translated into an even distribution across the experimental groups. Trust in science and research, however, was slightly higher than in the reference sample (63.5% vs. 56%; Wissenschaft im Dialog and Kantar, 2023), which may be because it was obvious that the study was conducted by a university. The content of each pair of videos was identical, and all subjects completed the same quiz questions on all topics to ensure that effects do not result from prior knowledge differences. Furthermore, the video host was a student; participants in this study were not already familiar with the videos or had a (para‐)social relationship with the host.

However, there are several limitations of this study that need to be acknowledged. First, all videos addressed nutrition‐related topics. Although they focus on different subdisciplines of nutritional science and related subjects (e.g. agriculture and ecology in the climate video, physiology and medicine in the sugar video, behavioural science in the nudging video), they can still be considered relatively similar. Whether the findings translate to other sciences (e.g. physics) remains to be tested. Similarly, all three talking‐head videos featured the same presenter, a Caucasian woman in her twenties. Indeed, female and male science communicators may induce differential reactions (Dalyot et al., 2022); generalizability of the findings to presenters of other genders, ethnicities and age groups therefore remains to be tested. The same is true for the visual style of the videos: due to a lack of resources, only one style could be implemented, and further work is needed to test whether different visual styles may have differential effects.

Second, although we recruited a representative sample, the sample size was too small to test for subgroup differences, e.g. regarding socio‐economic status. Since low socio‐economic status groups are more difficult to reach through traditional science communication activities (c.f., Humm & Schrögel, 2020), it would be important to test whether the results indeed generalize to this group and other underserved audiences. Given that we used a panel provider to recruit the sample, it is furthermore to be expected that participants were relatively interested in research and science, or that science sceptics were underrepresented. Indeed, prior research suggests that typical samples recruited from various subject pools differ e.g. in belief in conspiracy theories (Douglas et al., 2023); however, we are unable to compare our sample, or the platform we used, to this data.

Third, Schneider et al. (2022) suggest age differences could influence perceived strength of social signals. The video host in this study is a young woman, potentially impacting the responses of the average participants, who are around 46 years old, compared to a younger group.

Fourth, the quiz we used to assess knowledge only assessed factual knowledge, but not deeper structural knowledge. Using the same videos as in Boy et al. (2020) and applying a concept mapping technique, Bucher et al. (2022) suggested that animation videos might be superior in conveying structural knowledge. They attributed this to a multimodal structure, created by separating spoken commentary or explanatory text and visualizations into two distinct reception channels. More research is needed to confirm this hypothesis. Moreover, differences in knowledge scores between topics suggest that quizzes differed in difficulty. While video formats were primarily compared within a topic (RQ 1), this inherent differences in difficulty somewhat complicated the comparisons of knowledge scores achieved across topics (RQ 2).

Finally, the present study focused on the effectiveness of videos to convey nutrition knowledge and content sharing. These are important, but by no means the only objectives that science communication may have. Future research therefore should test a broader range of outcomes, including attitudes and trust towards science or willingness to engage with science (Lungu et al., 2021; Ziegler & Fischer, 2020).

### Conclusions

Based on this online experiment with a representative German sample, we can conclude that both animated and talking‐head videos are suitable video formats for conveying nutrition knowledge in the context of science communication. Other considerations, such as skill and time as well as target group preferences, may thus guide format choice when creating science communication videos.

## AUTHOR CONTRIBUTIONS

**Clara L. Marx:** Conceptualization; methodology; writing – original draft; investigation; data curation; formal analysis; project administration; visualization. **Laura M. König:** Conceptualization; formal analysis; methodology; software; supervision; writing – review and editing.

## FUNDING INFORMATION

This study did not receive external funding.

## CONFLICT OF INTEREST STATEMENT

The authors declare no competing interests.

## ETHICS STATEMENT

The study received approval from the University of Bayreuth ethics committee.

## DECLARATION OF GENERATIVE AI AND AI‐ASSISTED TECHNOLOGIES IN THE WRITING PROCESS

No generative AI was used in preparing this work.

## PERMISSION TO REPRODUCE MATERIAL FROM OTHER SOURCES

Not applicable.

## CLINICAL TRIAL REGISTRATION

The study was preregistered prior to the start of data collection: https://aspredicted.org/P29_B8V. An amendment was preregistered at https://osf.io/4nqpg/.

## Supporting information


Data S1.

Tables S1–S3.

## Data Availability

Data and materials are available on the study's Open Science Framework page: https://osf.io/4u6bc/.
