# Supplementary material for: Comparing the effectiveness of animated videos and talking‐head videos in science communication
Source: Br J Health Psychol. 2025 Feb 20;30(1):e12786. doi: 10.1111/bjhp.12786 (PMC11840882; doi:10.1111/bjhp.12786)
Supplement: Supplementary file 1 — Data S1. [file BJHP-30-0-s001.pdf]

The questionnaire was administered in German. This English translation is for documentation purposes only.

I have read and understood the information. I agree to the storage of the collected data for scientific purposes. I am at least 18 years old.

The questionnaire was administered in German. This English translation is for documentation purposes only.

Personal information

Your Age	____ Years		
You are …	☐1 male	☐2 female	☐3 diverse

Your highest level of education

have (yet) no degree	☐1
Primary, secondary, middle school graduation	☐2
Intermediate maturity, secondary school graduation	☐3
Polytechnic high school graduation	☐4
Graduation from a (vocational) technical school	☐5
High school diploma	☐6
Other degree, namely: ____________________	☐7

Your highest level of vocational training

have (yet) no completed vocational training	☐1
Apprenticeship	☐2
Technical secondary school	☐3
Vocational school	☐4
University of Applied Sciences	☐5
University	☐6
Doctorate	☐7
Other degree, namely: ____________________	☐8

Currently, you are …

employed	☐1
in training, retraining or studying	☐2
unemployed	☐3
retired	☐4
housewife or househusband only	☐5

If you are studying or have studied: Please specify your field of study.	____

The questionnaire was administered in German. This English translation is for documentation purposes only.

If you are employed: Please specify your job title. ______________________________

The questionnaire was administered in German. This English translation is for documentation purposes only.

Questions about Interest, Attitudes, and Media Usage

How interested are you in the following topics?

	not at all	rather not	neutral	rather	a lot
Nutrition	☐1	☐2	☐3	☐4	☐5
Health	☐1	☐2	☐3	☐4	☐5
Sustainability	☐1	☐2	☐3	☐4	☐5

How much do you trust ...?

	not	rather not	undecided	rather	fully
Science and research	☐1	☐2	☐3	☐4	☐5
Scientists at universities and public research institutions	☐1	☐2	☐3	☐4	☐5
Scientists in industry and economy	☐1	☐2	☐3	☐4	☐5

How big is your interest in science and research?

very low	rather low	half and half	rather high	very high
☐1	☐2	☐3	☐4	☐5

How often do you use the following video formats?

	never	once a month or less	several times a month	once a week	several times a week	(almost) daily
Videos with presenter	☐1	☐2	☐3	☐4	☐5	☐6
Animated videos	☐1	☐2	☐3	☐4	☐5	☐6

How often do you use the following video formats to inform yourself about nutritional topics (excluding recipes)?

	never	once a month or less	several times a month	once a week	several times a week	(almost) daily
Videos with presenter	☐1	☐2	☐3	☐4	☐5	☐6
Animated videos	☐1	☐2	☐3	☐4	☐5	☐6

The questionnaire was administered in German. This English translation is for documentation purposes only.

Exposition

[Condition A1-A2: Talking-head or animated video about nutrition and climate]
[Condition B1-B2: Talking-head or animated video about sugar in foods]
[Condition C1-C2: Talking-head or animated video about nudging]

The questionnaire was administered in German. This English translation is for documentation purposes only.

[Condition A1]

Talking-Head-Video on the topic of "Nutrition and Climate"

Below you will find a video on the topic of "Nutrition and Climate".

Please watch this carefully and calmly.

https://youtu.be/hJgppG1crAI

The questionnaire was administered in German. This English translation is for documentation purposes only.

Your Video Review

Please provide your review of the video below. There is no right or wrong answer. Your personal opinion matters!

The video was …

bad	☐1	☐2	☐3	☐4	☐5	☐6	good
not at all interesting	☐1	☐2	☐3	☐4	☐5	☐6	very interesting
not at all informative	☐1	☐2	☐3	☐4	☐5	☐6	very informative
poor quality	☐1	☐2	☐3	☐4	☐5	☐6	high quality
very poorly researched	☐1	☐2	☐3	☐4	☐5	☐6	very well researched
very boring	☐1	☐2	☐3	☐4	☐5	☐6	very exciting
sleep-inducing	☐1	☐2	☐3	☐4	☐5	☐6	activating
unpleasant	☐1	☐2	☐3	☐4	☐5	☐6	pleasant
very difficult to understand	☐1	☐2	☐3	☐4	☐5	☐6	very easy to understand
not at all appealing	☐1	☐2	☐3	☐4	☐5	☐6	very appealing
confusing	☐1	☐2	☐3	☐4	☐5	☐6	clear

The contents were ...

very unreliable	☐1	☐2	☐3	☐4	☐5	☐6	very reliable
very unprofessional	☐1	☐2	☐3	☐4	☐5	☐6	very professional
little trustworthy	☐1	☐2	☐3	☐4	☐5	☐6	very trustworthy

Would you...

	no, definitely not						yes, definitely
mention the information contained in the text in a personal conversation with others?	☐1	☐2	☐3	☐4	☐5	☐6	☐7
share the information with others on social media (e.g. X, Facebook, WhatsApp)?	☐1	☐2	☐3	☐4	☐5	☐6	☐7

The questionnaire was administered in German. This English translation is for documentation purposes only.

If you read this sentence, please select "no, definitely not".	☐1	☐2	☐3	☐4	☐5	☐6	☐7

The questionnaire was administered in German. This English translation is for documentation purposes only.

[Condition A2]

Animated video on the topic "Nutrition and Climate"

Below, you will see a video on the topic of "Nutrition and Climate".

Please watch this attentively and calmly.

https://youtu.be/nTC3hhYWyso

The questionnaire was administered in German. This English translation is for documentation purposes only.

Your Video Review

Please provide your review of the video below. There is no right or wrong answer. Your personal opinion matters!

The video was …

bad	☐1	☐2	☐3	☐4	☐5	☐6	good
not at all interesting	☐1	☐2	☐3	☐4	☐5	☐6	very interesting
not at all informative	☐1	☐2	☐3	☐4	☐5	☐6	very informative
poor quality	☐1	☐2	☐3	☐4	☐5	☐6	high quality
very poorly researched	☐1	☐2	☐3	☐4	☐5	☐6	very well researched
very boring	☐1	☐2	☐3	☐4	☐5	☐6	very exciting
sleep-inducing	☐1	☐2	☐3	☐4	☐5	☐6	activating
unpleasant	☐1	☐2	☐3	☐4	☐5	☐6	pleasant
very difficult to understand	☐1	☐2	☐3	☐4	☐5	☐6	very easy to understand
not at all appealing	☐1	☐2	☐3	☐4	☐5	☐6	very appealing
confusing	☐1	☐2	☐3	☐4	☐5	☐6	clear

The contents were ...

very unreliable	☐1	☐2	☐3	☐4	☐5	☐6	very reliable
very unprofessional	☐1	☐2	☐3	☐4	☐5	☐6	very professional
little trustworthy	☐1	☐2	☐3	☐4	☐5	☐6	very trustworthy

Would you...

	no, definitely not						yes, definitely
mention the information contained in the text in a personal conversation with others?	☐1	☐2	☐3	☐4	☐5	☐6	☐7
share the information with others on social media (e.g. X, Facebook, WhatsApp)?	☐1	☐2	☐3	☐4	☐5	☐6	☐7

The questionnaire was administered in German. This English translation is for documentation purposes only.

If you read this sentence, please select "no, definitely not".	☐1	☐2	☐3	☐4	☐5	☐6	☐7

The questionnaire was administered in German. This English translation is for documentation purposes only.

[Condition B1]

Talking-Head-Video on the topic "Sugar in Food"

Below you will find a video on the topic "Sugar in Food".

Please watch this calmly and listen carefully.

https://youtu.be/nZAAucyiKYQ

The questionnaire was administered in German. This English translation is for documentation purposes only.

Your Video Review

Please provide your review of the video below. There is no right or wrong answer.
Your personal opinion matters!

The video was …

bad	☐1	☐2	☐3	☐4	☐5	☐6	good
not at all interesting	☐1	☐2	☐3	☐4	☐5	☐6	very interesting
not at all informative	☐1	☐2	☐3	☐4	☐5	☐6	very informative
poor quality	☐1	☐2	☐3	☐4	☐5	☐6	high quality
very poorly researched	☐1	☐2	☐3	☐4	☐5	☐6	very well researched
very boring	☐1	☐2	☐3	☐4	☐5	☐6	very exciting
sleep-inducing	☐1	☐2	☐3	☐4	☐5	☐6	activating
unpleasant	☐1	☐2	☐3	☐4	☐5	☐6	pleasant
very difficult to understand	☐1	☐2	☐3	☐4	☐5	☐6	very easy to understand
not at all appealing	☐1	☐2	☐3	☐4	☐5	☐6	very appealing
confusing	☐1	☐2	☐3	☐4	☐5	☐6	clear

The contents were ...

very unreliable	☐1	☐2	☐3	☐4	☐5	☐6	very reliable
very unprofessional	☐1	☐2	☐3	☐4	☐5	☐6	very professional
little trustworthy	☐1	☐2	☐3	☐4	☐5	☐6	very trustworthy

Would you...

	no, definitely not						yes, definitely
mention the information contained in the text in a personal conversation with others?	☐1	☐2	☐3	☐4	☐5	☐6	☐7
share the information with others on social media (e.g. X, Facebook, WhatsApp)?	☐1	☐2	☐3	☐4	☐5	☐6	☐7

The questionnaire was administered in German. This English translation is for documentation purposes only.

If you read this sentence, please select "no, definitely not".	☐1	☐2	☐3	☐4	☐5	☐6	☐7

The questionnaire was administered in German. This English translation is for documentation purposes only.

[Condition B2]

Animated Video on the Topic "Sugar in Foods"

Below you will find a video on the topic "Sugar in Foods".

Please watch this quietly and listen carefully.

https://youtu.be/NjhcUpJo7CM

The questionnaire was administered in German. This English translation is for documentation purposes only.

Your Video Review

Please provide your review of the video below. There is no right or wrong answer.
Your personal opinion matters!

The video was …

bad	☐1	☐2	☐3	☐4	☐5	☐6	good
not at all interesting	☐1	☐2	☐3	☐4	☐5	☐6	very interesting
not at all informative	☐1	☐2	☐3	☐4	☐5	☐6	very informative
poor quality	☐1	☐2	☐3	☐4	☐5	☐6	high quality
very poorly researched	☐1	☐2	☐3	☐4	☐5	☐6	very well researched
very boring	☐1	☐2	☐3	☐4	☐5	☐6	very exciting
sleep-inducing	☐1	☐2	☐3	☐4	☐5	☐6	activating
unpleasant	☐1	☐2	☐3	☐4	☐5	☐6	pleasant
very difficult to understand	☐1	☐2	☐3	☐4	☐5	☐6	very easy to understand
not at all appealing	☐1	☐2	☐3	☐4	☐5	☐6	very appealing
confusing	☐1	☐2	☐3	☐4	☐5	☐6	clear

The contents were ...

very unreliable	☐1	☐2	☐3	☐4	☐5	☐6	very reliable
very unprofessional	☐1	☐2	☐3	☐4	☐5	☐6	very professional
little trustworthy	☐1	☐2	☐3	☐4	☐5	☐6	very trustworthy

Would you...

	no, definitely not						yes, definitely
mention the information contained in the text in a personal conversation with others?	☐1	☐2	☐3	☐4	☐5	☐6	☐7
share the information with others on social media (e.g. X, Facebook, WhatsApp)?	☐1	☐2	☐3	☐4	☐5	☐6	☐7

The questionnaire was administered in German. This English translation is for documentation purposes only.

If you read this sentence, please select "no, definitely not".	☐1	☐2	☐3	☐4	☐5	☐6	☐7

The questionnaire was administered in German. This English translation is for documentation purposes only.

[Condition C1]

Talking-Head-Video on the topic of "Nudging"

Below you will see a video on the topic of "Nudging".

Please watch this calmly and listen attentively.

https://youtu.be/AS3dZKMANUM

The questionnaire was administered in German. This English translation is for documentation purposes only.

Your Video Review

Please provide your review of the video below. There is no right or wrong answer. Your personal opinion matters!

The video was …

bad	☐1	☐2	☐3	☐4	☐5	☐6	good
not at all interesting	☐1	☐2	☐3	☐4	☐5	☐6	very interesting
not at all informative	☐1	☐2	☐3	☐4	☐5	☐6	very informative
poor quality	☐1	☐2	☐3	☐4	☐5	☐6	high quality
very poorly researched	☐1	☐2	☐3	☐4	☐5	☐6	very well researched
very boring	☐1	☐2	☐3	☐4	☐5	☐6	very exciting
sleep-inducing	☐1	☐2	☐3	☐4	☐5	☐6	activating
unpleasant	☐1	☐2	☐3	☐4	☐5	☐6	pleasant
very difficult to understand	☐1	☐2	☐3	☐4	☐5	☐6	very easy to understand
not at all appealing	☐1	☐2	☐3	☐4	☐5	☐6	very appealing
confusing	☐1	☐2	☐3	☐4	☐5	☐6	clear

The contents were ...

very unreliable	☐1	☐2	☐3	☐4	☐5	☐6	very reliable
very unprofessional	☐1	☐2	☐3	☐4	☐5	☐6	very professional
little trustworthy	☐1	☐2	☐3	☐4	☐5	☐6	very trustworthy

Would you...

	no, definitely not						yes, definitely
mention the information contained in the text in a personal conversation with others?	☐1	☐2	☐3	☐4	☐5	☐6	☐7
share the information with others on social media (e.g. X, Facebook, WhatsApp)?	☐1	☐2	☐3	☐4	☐5	☐6	☐7

The questionnaire was administered in German. This English translation is for documentation purposes only.

If you read this sentence, please select "no, definitely not".	☐1	☐2	☐3	☐4	☐5	☐6	☐7

The questionnaire was administered in German. This English translation is for documentation purposes only.

[Condition C2]

Animated video on the topic of "Nudging"

Below you will see a video on the topic of "Nudging".

Please watch this calmly and listen carefully.

https://youtu.be/wdmXoh-bwt8

The questionnaire was administered in German. This English translation is for documentation purposes only.

Your Video Review

Please provide your review of the video below. There is no right or wrong answer. Your personal opinion matters!

The video was …

bad	☐1	☐2	☐3	☐4	☐5	☐6	good
not at all interesting	☐1	☐2	☐3	☐4	☐5	☐6	very interesting
not at all informative	☐1	☐2	☐3	☐4	☐5	☐6	very informative
poor quality	☐1	☐2	☐3	☐4	☐5	☐6	high quality
very poorly researched	☐1	☐2	☐3	☐4	☐5	☐6	very well researched
very boring	☐1	☐2	☐3	☐4	☐5	☐6	very exciting
sleep-inducing	☐1	☐2	☐3	☐4	☐5	☐6	activating
unpleasant	☐1	☐2	☐3	☐4	☐5	☐6	pleasant
very difficult to understand	☐1	☐2	☐3	☐4	☐5	☐6	very easy to understand
not at all appealing	☐1	☐2	☐3	☐4	☐5	☐6	very appealing
confusing	☐1	☐2	☐3	☐4	☐5	☐6	clear

The contents were ...

very unreliable	☐1	☐2	☐3	☐4	☐5	☐6	very reliable
very unprofessional	☐1	☐2	☐3	☐4	☐5	☐6	very professional
little trustworthy	☐1	☐2	☐3	☐4	☐5	☐6	very trustworthy

Would you...

	no, definitely not						yes, definitely
mention the information contained in the text in a personal conversation with others?	☐1	☐2	☐3	☐4	☐5	☐6	☐7
share the information with others on social media (e.g. X, Facebook, WhatsApp)?	☐1	☐2	☐3	☐4	☐5	☐6	☐7

The questionnaire was administered in German. This English translation is for documentation purposes only.

If you read this sentence, please select "no, definitely not".	☐1	☐2	☐3	☐4	☐5	☐6	☐7

The questionnaire was administered in German. This English translation is for documentation purposes only.

Questions on the Topic of Nutrition

To conclude the study, we would like to ask you a few questions on various nutrition-related topics.

**Where is the climate-damaging methane gas CH_4 produced in agriculture?**	
In the keeping of ruminants and in rice cultivation	☐1
In the keeping of ruminants	☐2
In rice cultivation	☐3
In none of the mentioned types of production	☐4
Don‘t know	☐5

**If the wealth of the world's population grows, the demand for meat...**	
Increases	☐1
Decreases	☐2
Does not change	☐3
Don‘t know	☐4

**What do CO2 equivalents stand for?**	
They indicate the amount of CO2 produced by a certain activity	☐1
They indicate the amount of CO2 that has the same greenhouse potential as the actual mixture of greenhouse gases emitted	☐2
They indicate the amount of CO2 required to produce one kilogram of a food product	☐3
Don't know	☐4

**Arrange the products so that the least climate-damaging product receives the smallest number (1) and the most damaging product the largest number (3).**	
Milk	______
Pork	______
Beef	______

**How many liters of milk are needed to produce one packet of butter?**	
0,5L	☐1
1L	☐2
2L	☐3
4,5L	☐4

The questionnaire was administered in German. This English translation is for documentation purposes only.

Don't know	☐5

Which actor in the food value chain causes the largest proportion of wasted food?

Private households	☐1
Trade	☐2
Processing industry	☐3
Gastronomy	☐4
Don't know	☐5

When buying tomatoes of which cultivation method are on average more greenhouse gas emissions produced?

Cultivation in unheated greenhouse in Germany	☐1
Open field tomatoes from Spain	☐2
The greenhouse gas emissions are almost the same	☐3
Don't know	☐4

By how much do the annual greenhouse gas emissions caused by agriculture increase by 2050 according to the World Climate Council if the current development continues?

10-20 %	☐1
30-40 %	☐2
50-60 %	☐3
70-80 %	☐4
Don't know	☐5

The questionnaire was administered in German. This English translation is for documentation purposes only.

Questions on the Topic of Sugar

To conclude the study, we would like to ask you a few questions on various nutrition-related topics.

According to the recommendations of the World Health Organization, what percentage of daily energy intake should come from free sugars?

6 %	☐1
8 %	☐2
10 %	☐3
12 %	☐4
Don't know	☐5

Is there a proven link between the consumption of sugar-sweetened beverages and the development of obesity and an increased risk of other chronic diseases?

Yes, studies have proven this.	☐1
The studies on this are unclear.	☐2
No, a link cannot be proven.	☐3
Don't know	☐4

According to estimates by the World Health Organization, what is the reduction in sugar consumption that can be achieved with a 20 percent sugar tax?

5 %	☐1
20 %	☐2
40 %	☐3
Sugar taxes cannot achieve a long-term reduction in consumption.	☐4
Don't know	☐5

Why can you get cravings after eating foods high in sugar?

Because blood sugar levels rise, which causes insulin levels to drop.	☐1
Because insulin levels rise, which causes blood sugar levels to drop.	☐2
Because both blood sugar levels and insulin levels drop.	☐3
None of the above statements are true.	☐4
Don't know	☐5

Why is fructose from unprocessed fruit not counted as free sugars, even though it is counted in processed fruit such as juices?

Because unprocessed fruit contains fiber, which has a positive effect on the absorption of fructose.	☐1

The questionnaire was administered in German. This English translation is for documentation purposes only.

Because fruit juices always have artificial fructose added to make them sweeter.	☐2
Because free sugars only occur in liquids.	☐3
None of the above statements are true.	☐4
Don't know	☐5

Which of the mentioned terms refer to sugar or other sweetening ingredients found in food? (Multiple answers can be chosen.)

Barley malt extract	☐1
Maltodextrin	☐2
Glucose-fructose syrup	☐3
Saccharin	☐4
Caramel color	☐5
Don't know	☐6

Why is sugar also used in savory foods such as ketchup?

Because it enhances the color of the food.	☐1
Because it is cheaper than salt.	☐2
Because it serves as a flavor carrier	☐3
None of the above statements are true..	☐4
Don't know	☐5

Which of the mentioned foods is not one of the most common sources of free sugars in Germany?

Dried fruit	☐1
Ketchup	☐2
Muesli	☐3
Fruit yogurt	☐4
Fruit juice	☐5
Don't know	☐6

The questionnaire was administered in German. This English translation is for documentation purposes only.

Questions on the Topic of Nudging

To conclude the study, we would like to ask you a few questions on various nutrition-related topics.

What percentage of annual deaths in Germany can be attributed to poor nutrition?

2%	☐1
5%	☐2
10%	☐3
20%	☐4
Don't know	☐5

Measures restricting the sale of unhealthy snacks at kiosks and smaller shops are ... in the population

Not accepted at all	☐1
Rather not accepted	☐2
Rather accepted	☐3
Accepted	☐4
Don't know	☐5

Which measures correspond to the concept of "nudging"? (Multiple answers can be chosen.)

Placement of water bottles at eye level and lemonade on the bottom shelf in a beverage refrigerator	☐1
Offering salad as a standard side dish in cafeterias. Whoever wants fries instead has to order explicitly	☐2
Offering salad as the only side dish in cafeterias	☐3
Chocolate bars are removed from the offer	☐4
Don't know	☐5

What is frequently criticized about nudging? Critics say that...

it restricts people's self-determination.	☐1
healthy food is more expensive which results in higher prices for the customers.	☐2
it is rejected by too many people.	☐3
the measures usually lead to customers eating elsewhere.	☐4
Don't know	☐5

How can healthy eating be most effectively supported by nudging?

The questionnaire was administered in German. This English translation is for documentation purposes only.

More healthier options are provided. These are placed so they are easily visible and accessible.	☐1
Signboards with information about healthy foods are set up.	☐2
The nutritional information of the dishes are provided.	☐3
References to the effects of consumption are mentioned (e.g. "invigorating").	☐4
Don't know	☐5

Why does nudging work?

Because we often don't think too much about possible options and simply choose the first or usual option.	☐1
Because we like it when we can retain the freedom of choice - unlike sales bans for example.	☐2
Because it shows us the pros and cons of all available options so we can make rational decisions.	☐3
Don't know	☐4

In a study, ...% of respondents agreed with the statement: "People can be manipulated by nudging"

15% of respondents agreed.	☐1
20% of respondents agreed.	☐2
49% of respondents agreed.	☐3
70% of respondents agreed.	☐4
Don't know	☐5

What uncertainties still exist with nudging? (Multiple answers possible)

It is not clear whether the effects can be transferred to different settings (cafeteria/home).	☐1
It is not clear whether the measures show long-term effects.	☐2
It is not clear whether nudging works for everyone.	☐3
None of the statements apply.	☐4
Don't know	☐5

The questionnaire was administered in German. This English translation is for documentation purposes only.

Thank you very much for participating in our study.
