# Supplementary material for: Comparing the effectiveness of animated videos and talking‐head videos in science communication
Source: Br J Health Psychol. 2025 Feb 20;30(1):e12786. doi: 10.1111/bjhp.12786 (PMC11840882; doi:10.1111/bjhp.12786)
Supplement: Supplementary file 2 — Tables S1–S3. [file BJHP-30-0-s002.docx]

**Table S1.** Links to video material used in the study.

| Condition | Talking head | Animated |
| --- | --- | --- |
| Climate | <https://youtu.be/hJgppG1crAI> | <https://youtu.be/nTC3hhYWyso> |
| Sugar | <https://youtu.be/nZAAucyiKYQ> | <https://youtu.be/NjhcUpJo7CM> |
| Nudging | <https://youtu.be/AS3dZKMANUM> | <https://youtu.be/wdmXoh-bwt8> |

**Table S2.** Demographic characteristics per group.

|  |  | Climate | | Sugar | | Nudging | | Result of statistical test |
| --- | --- | --- | --- | --- | --- | --- | --- | --- |
|  |  | Talking-head | Animated | Talking-head | Animated | Talking-head | Animated |  |
| Age (M, SD) |  | 46.25, 15.40 | 45.24, 16.02 | 43.82, 16.52 | 49.05,  16.51 | 48.95, 16.51 | 45.47,  14.57 | *F*(5, 352) = 1.09, *p* = .366 |
| Gender (% per category) | Men | 50.8 | 45.8 | 47.5 | 59.3 | 51.7 | 45.0 | Χ²(*df*=10) = 8.45, *p* = .585 |
|  | Women | 47.5 | 54.2 | 52.5 | 40.7 | 48.3 | 55.0 |  |
|  | Diverse | 1.7 | 0.0 | 0.0 | 0.0 | 0.0 | 0.0 |  |
| Education (M, SD) |  | 13.85,  2.85 | 14.93,  2.65 | 14.16,  3.06 | 14.23,  2.42 | 13.45,  2.82 | 14.23,  2.59 | *F*(5, 352) = 1.91, *p* = .092 |
| Employment status (% per category) | Employed | 50.8 | 66.1 | 52.5 | 57.6 | 51.7 | 68.3 | Χ²(*df*=20) = 15.81, *p* = .728 |
|  | In training/ in school | 6.8 | 6.8 | 8.2 | 6.8 | 8.3 | 8.3 |  |
|  | Unemployed | 5.1 | 5.1 | 11.5 | 8.5 | 6.7 | 3.3 |  |
|  | Retired | 25.4 | 16.9 | 21.3 | 25.4 | 25.0 | 13.3 |  |
|  | Homemaker | 11.9 | 5.1 | 6.6 | 1.7 | 8.3 | 6.7 |  |
| Interest in nutrition (M, SD) |  | 3.68,  1.21 | 3.90,  1,16 | 3.62,  1.11 | 3.85,  1.00 | 3.83,  1.08 | 3.95,  1.00 | *F*(5, 352) = 0.82, *p* = .537 |
| Interest in health (M, SD) |  | 4.02,  1.08 | 4.07,  1.03 | 3.75,  1.22 | 4.12,  1.00 | 3.92,  1.18 | 4.07,  0.90 | *F*(5, 352) = 0.94, *p* = .453 |
| Interest in sustainability (M, SD) |  | 3.68,  1.12 | 3.46,  1.30 | 3.59,  1.23 | 3.68,  1.06 | 3.48,  1.19 | 3.47,  1.33 | *F*(5, 352) = 0.44, *p* = .822 |
| Trust in science and research (M, SD) |  | 3.68,  1.04 | 3.81,  0.90 | 3.48,  1.26 | 3.54,  1.02 | 3.57,  1.03 | 3.72,  1.08 | *F*(5, 352) = 0.65, *p* = .659 |
| Trust in universities and public research institutions (M, SD) |  | 3.51,  1.09 | 3.47,  0.90 | 3.08,  1.20 | 3.27,  0.98 | 3.07,  1.09 | 3.27,  1.02 | *F*(5, 352) = 1.15, *p* = .332 |
| Trust in scientists in industry (M, SD) |  | 3.68,  1.04 | 3.81,  0.90 | 3.61,  1.12 | 3.56,  1.02 | 3.57,  1.06 | 3.77,  0.95 | *F*(5, 352) = 1.25, *p* = .285 |
| Frequency of watching talking-head videos (% per category) | never | 42.4 | 37.3 | 39.3 | 37.3 | 48.3 | 46.7 | Χ²(*df*=30) = 33.54, *p* = .300 |
|  | Once a month or less | 28.8 | 22.0 | 13.1 | 23.7 | 13.3 | 18.3 |  |
|  | Several times a month | 13.6 | 15.3 | 11.5 | 16.9 | 16.7 | 6.7 |  |
|  | Once a week | 5.1 | 11.9 | 13.1 | 6.8 | 6.7 | 3.3 |  |
|  | Several times a week | 3.4 | 10.2 | 16.4 | 10.2 | 8.3 | 15.0 |  |
|  | (almost) daily | 6.8 | 3.4 | 6.6 | 5.1 | 6.7 | 6.7 |  |
| Frequency of watching animated videos (% per category) | never | 37.3 | 35.6 | 37.7 | 27.1 | 46.7 | 36.7 | Χ²(*df*=30) = 28.07, *p* = 0.567 |
|  | Once a month or less | 30.5 | 20.3 | 16.4 | 32.2 | 15.0 | 35.0 |  |
|  | Several times a month | 15.3 | 22.0 | 21.3 | 16.9 | 18.3 | 6.7 |  |
|  | Once a week | 6.8 | 8.5 | 8.2 | 10.2 | 5.0 | 6.7 |  |
|  | Several times a week | 6.8 | 6.8 | 4.9 | 10.2 | 8.3 | 11.7 |  |
|  | (almost) daily | 3.4 | 5.1 | 9.8 | 3.4 | 5.0 | 3.3 |  |

**Table S3.** Analysis of effects in Bayesian mixed ANOVAs with BF_01_.

| Effects | P(incl) | P(incl\|data) | BF_excl_ | Interpretation (Jeffreys, 1961; c.f. Jarosz and Wiley, 2014) |
| --- | --- | --- | --- | --- |
| **RQ1, H1.1, outcome variable knowledge score** | | | | |
| Topic | 0.60 | 0.09 | 15.90 | Strong |
| Medium | 0.60 | 0.11 | 11.71 | Strong |
| Topic x Medium | 0.20 | 0.01 | 52.90 | Very strong |
| **RQ1, H1.2, outcome variable verbal knowledge score** | | | | |
| Topic | 0.60 | 0.08 | 18.56 | Strong |
| Medium | 0.60 | 0.15 | 8.50 | Substantial |
| Topic x Medium | 0.20 | 0.00 | 103.77 | Decisive |
| **RQ1, H1.3, outcome variable numerical knowledge score** | | | | |
| Topic | 0.60 | 0.54 | 1.30 | Anecdotal |
| Medium | 0.60 | 0.13 | 9.82 | Substantial |
| Topic x Medium | 0.20 | 0.02 | 16.58 | Strong |
| **RQ1, H1.4, outcome variable inferred knowledge score** | | | | |
| Topic | 0.60 | 0.85 | 0.27 |  |
| Medium | 0.60 | 0.15 | 8.30 | Substantial |
| Topic x Medium | 0.20 | 0.03 | 7.93 | Substantial |
| **RQ2, H2.1, outcome variable knowledge score** | | | | |
| Knowledge | 0.74 | 1.00 | 0.00 |  |
| Topic | 0.74 | 1.00 | 0.00 |  |
| Medium | 0.74 | 0.17 | 14.18 | Strong |
| Knowledge x Topic | 0.32 | 1.00 | 0.00 |  |
| Knowledge x Medium | 0.32 | 0.02 | 23.84 | Strong |
| Topic x Medium | 0.32 | 0.03 | 17.14 | Strong |
| Knowledge x Topic x Medium | 0.05 | < 0.01 | 380.48 | Decisive |
| **RQ2, H2.2, outcome variable numerical knowledge** | | | | |
| Knowledge | 0.74 | 1.00 | 0.00 |  |
| Topic | 0.74 | 1.00 | 0.00 |  |
| Medium | 0.74 | 0.29 | 6.86 | Substantial |
| Knowledge x Topic | 0.32 | 1.00 | 0.00 |  |
| Knowledge x Medium | 0.32 | 0.02 | 19.70 | Strong |
| Topic x Medium | 0.32 | 0.01 | 46.72 | Very strong |
| Knowledge x Topic x Medium | 0.05 | 0.00 | 2398.61 | Decisive |
| **RQ2, H2.3, outcome variable verbal knowledge** | | | | |
| Knowledge | 0.74 | 1.00 | 0.00 |  |
| Topic | 0.74 | 1.00 | 0.00 |  |
| Medium | 0.74 | 0.11 | 21.75 | Strong |
| Knowledge x Topic | 0.32 | 1.00 | 0.00 |  |
| Knowledge x Medium | 0.32 | 0.00 | 102.36 | Decisive |
| Topic x Medium | 0.32 | 0.02 | 22.61 | Strong |
| Knowledge x Topic x Medium | 0.05 | 0.00 | 1519.82 | Decisive |
| **RQ2, H2.4, outcome variable inferred knowledge** | | | | |
| Knowledge | 0.74 | 1.00 | 0.00 |  |
| Topic | 0.74 | 1.00 | 0.00 |  |
| Medium | 0.74 | 0.12 | 21.35 | Strong |
| Knowledge x Topic | 0.32 | 1.00 | 0.00 |  |
| Knowledge x Medium | 0.32 | 0.01 | 100.67 | Decisive |
| Topic x Medium | 0.32 | 0.01 | 51.27 | Very strong |
| Knowledge x Topic x Medium | 0.05 | 0.00 | 6151.13 | Decisive |

References

Jarosz, A. F., & Wiley, J. (2014). What are the odds? A practical guide to computing and reporting Bayes factors. *The Journal of Problem Solving*, *7*(1), 2.

Jeffreys, H. (1961). *Theory of probability* (3rd Ed.). Oxford, UK: Oxford University Press
